# Trust and Engagement on Twitter During the Management of COVID-19 Pandemic: The Effect of Gender and Position

**DOI:** 10.3389/fsoc.2022.811589

**Published:** 2022-04-04

**Authors:** Samira Yousefinaghani, Rozita Dara, Melissa MacKay, Andrew Papadopoulos, Shayan Sharif

**Affiliations:** ^1^School of Computer Science, University of Guelph, Guelph, ON, Canada; ^2^Department of Population Medicine, University of Guelph, Guelph, ON, Canada; ^3^Department of Pathobiology, University of Guelph, Guelph, ON, Canada

**Keywords:** trust, public health, government, COVID-19 pandemic, Twitter, health leaders, political leaders

## Abstract

During the COVID-19 pandemic, health and political leaders have attempted to update citizens using Twitter. Here, we examined the difference between environments that social media has provided for male/female or health/political leaders to interact with people during the COVID-19 pandemic. The comparison was made based on the content of posts and public responses to those posts as well as user-level and post-level metrics. Our findings suggest that although health officers and female leaders generated more contents on Twitter, political leaders and male authorities were more active in building networks. Offensive language was used more frequently toward males than females and toward political leaders than health leaders. The public also used more appreciation keywords toward health leaders than politicians, while more judgmental and economy-related keywords were used toward politicians. Overall, depending on the gender and position of leaders, Twitter provided them with different environments to communicate and manage the pandemic.

## Introduction

As the world is contending with the COVID-19 pandemic, the public has been using social media, including Twitter as a source of information (Sattar and Arifuzzaman, 2021; Yousefinaghani et al., 2021). Twitter has emerged as a novel way of communication between leaders and the public during the COVID-19 crisis. Globally, crisis communication has been used to provide information and public health directives to reduce disease transmission (Jong, 2021). Effective crisis communication that helps maintain trust in leaders requires clear messages about the risks including compassion, empathy, and ongoing engagement with communities (Hyland-Wood et al., 2021).

Unfortunately, trust in authorities has declined globally, resulting from widespread distrust of social institutions and the “infodemic” or widespread mis- and dis-information that undermines official crisis messaging (Ahern and Loh, 2020). Now more than ever, effective leadership and communication with the public is extremely crucial. Support for policies and recommendations during a pandemic is difficult to achieve without trust in leading organizations, especially when individuals must make sacrifices for uncertain long-term benefits (Ahern and Loh, 2020).

Greater trust in political and public health leaders results in greater compliance with public health policies and control measures and thus, is a crucial aspect of overcoming COVID-19 (Devine et al., 2020). During a crisis, people tend to trust leaders who are focused on the relational aspects of managing the crisis (Post et al., 2019). Relational behaviors such as showing compassion and anticipating and managing the emotions of others during a crisis can help build and restore trust (Post et al., 2019). For that reason, there can be a “female leadership trust advantage” that sometimes occurs during a crisis (Post et al., 2019).

Studies indicated that women use social media more effectively than men and female leaders make social media a supportive network to connect with other female leaders (McGregor and Mourão, 2016; Hayat et al., 2017). Yarchi and Samuel-Azran (2018) compared the user engagement in social media for men and women politicians during an election campaign in Israel. This study found females generated more engagements in terms of likes and shares vs. male politicians, which led to the conclusion that the social media might help female politicians promote themselves and increase their social and political status.

On the other hand, there is a perception that men make better leaders than women in leadership research (Yarchi and Samuel-Azran, 2018). Hayat et al. (2017) indicated that leadership roles and conversations have been dominated by males in online social network discussions. Similarly, an analysis (Brandtzaeg, 2017) on Facebook concluded that males are more interested in political subjects while females are attracted to interpersonal issues.

Mertens et al. (2019) studied gender bias in digital communications during the German federal elections in 2017 and discovered that tweets in reply to female politicians were more personal than professional compared to tweets directed at males.

There is an increasing attention on the use of social media by the public and political election candidates. However, less is known about the existence of gender and position biases in the use of social media by political and public health leaders during emergency situations. Additionally, very few previous approaches combined sentiment analysis and engagement metrics to gain insight into biases toward health and political leaders on social media platforms.

The present study aimed to gain insight into the role of gender and the position of authorities in building users' trust and engagement through social media during the COVID19 pandemic in Canada and the United States. The main objectives were: (1) to quantify tweets sentiments and the measures of distrust and offense in tweets authored by and replied to leaders, and to further understand the role of gender and position in these measures; and, (2) to identify latent gender biases in terms of popularity metrics of leaders' accounts and communications by and directed at them. Previous studies indicated (Alrubaian et al., 2017; Arora et al., 2019) that sentiment and popularity-based features play a key role in identifying influential and credible users on social media platforms.

## Materials and Methods

**Figure 1:**
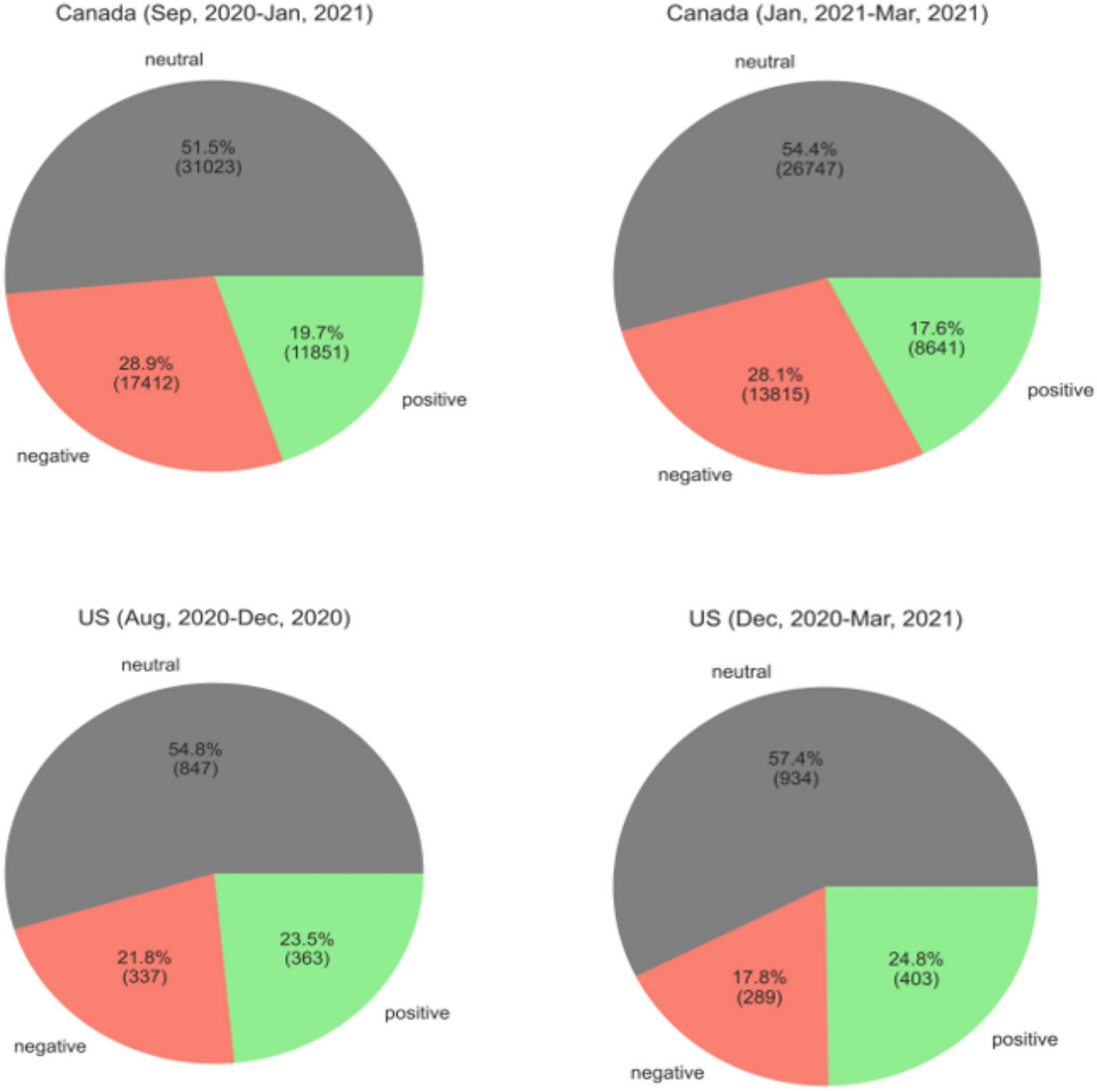
Sentiment analysis of tweets in reply to administrators during COVID-19 in Canada and the US before and after vaccination.

A flow of the methodological approach is shown in Figure 1, three types of data were collected from Twitter. Subsequently, we analyzed the collected data to extract or construct required features for comparing the groups of male/female or political/health officer authorities.

### Data Acquisition

The Twitter Premium Search API (Application Programming Interface) was used to collect English tweets containing COVID-19 related terms given in Table 1. Posts by 38 public health official and politician accounts from Canada and the US as well as replies to these posts were collected from January 1, 2020 to March 16, 2021.

**Table 1:** COVID-19 related terms used in Twitter API.

**Search terms**
COVID, COVID-19, COVID19, coronavirus, SARACoV2, pandemic, vaccine, vaccinate, travel, treatment, testing, stay home, staying home, stay at home, stay-at-home, gathering, and mask

In addition, we utilized a user lookup Twitter API to access engagement information of users, including the number of favorites, followers and friends. The number of favorites means the number of times each leader's account liked others' tweets. Followers of a user are Twitter users that follow a specific user while friends are Twitter users that a specific user follows.

In total, we collected 184 K tweets including direct posts (19 K) and replies (165 K) for 38 authorities from Canada and the US. Among these authorities, 17 were female and 21 were male, 13 were political leaders and 25 were health officials. Reply tweets were generated by 57,539 users. In the data table where we stored tweet, we specified different categories by adding several extra fields such as “gender,” “position,” “is reply,” and “is retweet.”

### Data Analysis

Male/Female and political/health leader groups were compared with regard to three sets of aspects including content-based, tweet-level and user-level features. We determined whether the feature samples fit a normal distribution using the “normaltest” function in the “Scipy” Python library scipy (Scipy, 2021). “normaltest” did not indicate a normal distribution for the samples. Hence, we chose to compare the groups of features using non-parametric MannWhitney U tests with a *p*-value < 0.05 as statistically significant.

### Content Analysis

#### Trust/Distrust Classification

To automatically determine the class labels (trust/distrust) of unlabelled response tweets, we adopted an expectation-maximization (EM) based semisupervised classifier (Nigam et al., 2006; Karimpour et al., 2012). EM is an iterative algorithm to maximize a posteriori estimation in datasets with both labeled and unlabeled data (Nigam et al., 2000). To avoid over-fitting of the model due to imbalanced data, we oversampled the minority class (trust) in the initial dataset. This was done by adding random tweets that demonstrated trust toward leaders. Tweets indicating appreciation, respect and prayers were labeled with “trust” (1 K) and those showing disrespect and ingratitude were labeled with “distrust” (26 K). Examples of tweets indicating distrust are given in Appendix Table 3. The output variables of data instances containing “trust” and “distrust” tweets were assigned with 0 and 1 values, respectively. To represent the text data in the learning model, we converted tweets into term frequency and inverse document frequency (TF-IDF) feature vectors.

The semi-supervised learning was performed using the Python “SKlearn” library (Scikit-Learn, 2021). The algorithm (EM) trained a classifier model with only labeled tweets, which then was used to assign labels to unlabeled tweets by calculating the expectation of missing values. We utilized the Naive Bayes model as the base classier, which is fast and commonly used in text classification. Subsequently, in an iterative process, a new classifier was trained using all tweets (originally labeled and unlabeled). To find the classifier parameters that maximize the likelihood of the data, EM uses hill-climbing optimization (Nigam et al., 2000). The average test performance was evaluated using 10-fold cross-validation, showing an accuracy of 74.34% and an F-score of 60.71%.

Finally, the proportion of distrust to total replies for each leader was calculated and used for comparisons. Subsequently, we constructed distrust measures by dividing the metrics of tweets with “distrust” labels by the total tweets.

#### Insulting Content

A collection of 11 keywords and phrases were chosen to identify offense in comments. Keywords such as “crap,” “stupid,” “liar” and other insulting keywords can show disrespect and distrust toward leaders. For each individual leader, we divided the offensive replies by the total replies that they received, which was then used to compare different groups of leaders. The complete list of keywords is given in Appendix Table 2.

#### Keyword Extraction

The Latent Dirichlet Allocation (Blei et al., 2003) topic model was adopted to find important keywords that the public have used in reply to leaders' tweets. The model assumes documents are a mixture of topics and a topic is a probability distribution of words. Considering only one topic in each group of leaders including male political, male health officer, female political and female health officer leaders, assuming one topic, the most probable words were found.

Sentiment Analysis: To understand how critical events such as vaccination rollout has influenced the sentiments of replies to administrators, the reply tweets were divided into two periods based on vaccination time for Canada and the US. The COVID-19 vaccination in Canada started from around January and for US around December. We performed data cleaning on the response tweets for about 3 months before and after the start of vaccination. For the cleaning process, we removed URLs, mentions, and non-UTF8 (UCS Transformation Format 8) characters, then we used VADER (Valence Aware Dictionary for Sentiment Reasoning) sentiment API (Hutto and Gilbert, 2014) to label tweets to positive, negative, and neutral classes.

### Tweet-Level Metrics

Engagement metrics can measure the influence of Twitter users and their connection with followers (Neiger et al., 2013). We extracted certain interaction metrics such as the number of tweets, favorites and the number of times a post was shared (re-tweeted) for both posts and replies authored over the duration of 1 year and 2 months. The present study considered only the direct tweets posted by leaders and did not include the posts that leaders re-tweeted.

### User-Level Metrics

In addition to the aforementioned features, the influence of a user can be assessed with other user-derived features such as the number of friends, followers and favorites. In the present study, the aforementioned user lookup data was used to pull out these features.

## Results

### Content Analysis of Replies

No significant difference was found between males and females for replies indicating distrust while a significant difference between political and health officer leaders was detected for favorites (Table 2). The proportion of favorites in response to political leaders was higher compared to health officer leaders [mean_favourites_ (health officers) = 0.938 and mean_favourites_ (political leaders) = 1.004]. Moreover, the difference between the number of replies to tweets posted by health officers and political leaders was marginally significant [*P*-value = 0.087; mean_#posts_ (health officers) = 0.977 and mean_#posts_ (political leaders) = 0.992].

**Table 2:** Distrust in replies of posts authored by health leaders.

**Features**	**Group**	**Mean**	**Median**	***P*-value**
#Posts	Male leaders	0.983	0.9930	0.421
	Female leaders	0.981	0.991	
	Political leaders	0.992	0.994	0.087
	Health officers	0.977	0.990	0.143
Favorites	Male leaders	0.999	0.999	
	Female leaders	0.916	0.999	
	Political leaders	1.004	1.000	0.048^*^
	Health officers	0.938	0.999	0.161
Retweets	Male leaders	0.953	1.000	
	Female leaders	0.878	0.999	
	Political leaders	0.922	1.002	0.235
	Health officers	0.915	0.999	

* *P < 0.05*.

In Table 3, the difference between the proportion of offensive replies to males and females was significant [mean_replies_(males) = 0.084, mean_replies_(females) = 0.058]. Moreover, the difference in offensive language toward political and health officer leaders was marginally significant (*P*-value = 0.051). The measure of offensive language calculated by the number of replies to political leaders was higher than that to health officer leaders [mean_replies_ (health officers) = 0.064 and mean_replies_ (political leaders) = 0.088]. Engagement metrics of replies did not have any effects on the difference between male/female authorities or political/health officer leaders.

**Table 3:** Offense in replies of posts authored by health leaders.

**Features**	**Group**	**Mean**	**Median**	***P*-value**
#Posts	Male leaders	0.084	0.088	0.047^*^
	Female leaders	0.058	0.077	
	Political leaders	0.088	0.103	0.051
	Health officers	0.064	0.079	
Favorites	Male leaders	0.591	0.704	0.392
	Female leaders	0.590	0.816	
	Political leaders	0.530	0.600	0.360
	Health officers	0.621	0.766	
Retweets	Male leaders	0.660	0.685	0.294
	Females	0.762	0.807	
	Political leaders	0.540	0.685	0.223
	Health officers	0.787	0.799	

* *P < 0.05*.

The most important keywords for each group of leaders are given in Table 4 in descending order. The public replies to male politicians included themes of restrictions, control measures and economy, while for male health officers the themes included restrictions, control measures and appreciation. On the other hand, female politicians received judgmental comments, while female health officers received comments about restrictions and appreciation.

**Table 4:** Comparing important keywords in replies to leaders.

**Group of leaders**	**Keywords**
Male political leaders	Lockdown, government, essential, job, economy, better, plan, wearing, numbers, business
Male health officer leaders	Numbers, restrictions, wearing, positive, mandatory, thanks, social, better, great, gathering, essential order, hard
Female political leaders	Explain, residents, compliance, judgment, transparency, evading, supposed, essential, recommendations, difficult, seniors, graduate, students
Female health officer leaders	Government, PCR, numbers, positive, restrictions, social, quarantine, better, app, children, thanks, job

### Sentiment Analysis

The results of comparing positive, negative, and neutral sentiments before and after vaccination in Canada and the US is given in Figure 1. Generally, neutral was the majority sentiment which is consistent with the results of a similar study in Japan (Niu et al., 2021). The negative sentiment was more in Canada and the positive sentiment was more in the US. In Canada, the number of positive and negative responses to leaders has decreased after vaccinations in January 2021 by a 1 and a 2%, respectively. However, the US has seen a 4% drop in negative and a 2% increase in positive responses. The difference between the periods was more considerable in the US and shows that after vaccination in December in the US, the comments in response to administrators tend to have more positive polarity.

### Tweet-Level Metrics

The average number of tweets posted by females was 576 compared to 248 tweets by males while the opposite case was true for replies to their posts where males received three times more replies than females. The number of posts/replies, likes and shares between compared groups did not show a significant difference (Tables 5, 6). This could be due to the relatively small sample sizes.

**Table 5:** Comparing users' engagement of authorities' posts.

**Features**	**Group**	**Mean**	**Median**	***P*-value**
#Posts	Male leaders	248.7	162.5	0.338
	Female leaders	576.357	172.5	
	Political leaders	273.416	173.0	0.454
	Health officers	463.25	162.0	
Favorites	Male leaders	210.24	84.71	0.108
	Female leaders	101.92	42.23	
	Political leaders	240.95	76.549	0.430
	Health officers	115.995	76.114	
Retweets	Male leaders	39.29	18.88	0.253
	Female leaders	26.84	14.34	
	Political leaders	41.617	12.643	0.430
	Health officers	29.180	21.284	

**Table 6:** Comparing users' engagement of replies to authorities' posts.

**Features**	**Gender**	**Mean**	**Median**	**Statistic**
#Posts	Male leaders	6,624.9	303.0	0.256
	Female leaders	1,903.7	141.0	
	Political leaders	10,701.0	325.5	0.215
	Health officers	1,514.29	303.0	
Favorites	Male leaders	1.755	1.483	0.242
	Female leaders	1.507	1.39	
	Political leaders	1.869	1.483	0.246
	Health officers	1.541	1.403	
Retweets	Male leaders	0.233	c0.193	0.398
	Female leaders	0.208	0.201	
	Political leaders	0.257	0.209	0.145
	Health officers	0.207	0.194	

### User-Level Metrics

No significant difference was found between the number of followers of each group of authorities (Table 7). However, the difference in the number of friends and favorites was significant. The results show that male authorities have followed more Twitter accounts. 

**Table 7:** Comparing accounts properties for male and female authorities.

**Features**	**Group**	**Mean**	**Median**	***P*-value**
Friends	Male leaders	723.238	593.0	0.029^*^
	Female leaders	440.647	184.0	
	Political leaders	948.384	924.0	0.004^**^
	Health officers	414.0	195.0	0.017^*^
Favorites	Male leaders	3,783.809	1,572.0	
	Female leaders	1,296.529	456.0	
	Political leaders	5,328.53	1,749.0	0.014^*^
	Health officers	1,296.529	629.0	
Followers	Male leaders	63,451.095	13,371.0	0.075
	Female leaders	37,015.470	5,357.0	
	Political leaders	94,014.84	15,036.0	0.074
	Health Officers	29,581.72	8,611.0	

* *P < 0.05*;

** *P < 0.01*.

(mean_friends_ = 6,624) and have liked more posts (mean_favourites_ = 1.75) than female authorities. Similarly, the number of friends and favorites for political leaders (mean_friends_ = 948, mean_favourites_ = 5,328) was higher compared to public health officials. Male and political leaders being more active on Twitter in terms of adding more friends and liking more posts can explain why male and political leaders' posts generated more user engagement in the aforementioned section.

One possible explanation for the patterns of male and political leaders being the same in the present study is that the majority of males were political leaders, and the majority of females were health officer leaders.

## Discussion

One of the contributions of the present study is a joint consideration of engagement and content-based measures to reveal gender and position effects on Twitter discussions. Supervised or unsupervised models were commonly used in the literature (Castillo et al., 2011; Golbeck et al., 2011). However, there is a lack of labeled data in real-world problems. To tackle this issue, we used a semi-supervised model to identify tweets indicating “trust” and “distrust”.

### Trust in Sources of Crisis Communication

A survey of 2,000 Canadians showed the provincial response to COVID-19 was ranked highest compared to federal and municipal responses. This may be related to the large presence of provincial public health officials providing updated risk information on the pandemic daily in the Canadian media landscape, such as the Provincial Health Officer for British Columbia Dr. Bonnie Henry (Waddell, 2020). However, elected officials, such as premiers, are distrusted, often due to the perception they are motivated by political interests over public wellbeing (Gesser-Edelsburg et al., 2014). The current research saw higher distrust in replies to political leaders and higher offensive responses, which may be due to the underlying distrust in general for elected officials. Canadians also reported they most trust public health officials to provide credible information during the COVID-19 pandemic (Waddell, 2020), indicating they trust those with subject matter expertise to provide the most accurate information and effectively manage the risk. Similarly, a study in the United States of America found that federal and local public health were the most trusted sources of COVID-19 information compared to the White House officials (Fridman et al., 2020). Generally speaking, during infectious disease outbreaks, the public has higher trust in public health officials compared to elected officials and the media (Alsulaiman, 2018). Higher trust in public health leaders may account for less distrust and offense found in replies to their tweets.

### Trust in Male vs. Female Leaders

There has been much debate in research and the media regarding male vs. female leadership. Crises with low uncertainty in terms of long-term consequences have been found to benefit from female leaders with strong relational skills (Post et al., 2019). Relational skills, such as interpersonal communication, have been found to enhance trust and positively impact desired behaviors (Post et al., 2019). With regards to COVID-19, the debate has remained active. One study explored the relationship between COVID-19 related deaths and political gender leadership and found lower reported fatality rates in countries led by women, although not statistically significant (Windsor et al., 2020). The study notes the countries with female leaders managing COVID-19 well are often economically advantaged and often represent countries that have advanced social cultural and political norms and values, which likely contributes to the lower death rate (Windsor et al., 2020). Similarly, a study found states in the U.S.A. with women governors had fewer deaths than those governed by males (Sergent and Stajkovic, 2020). Findings suggest that public health directives, such as stay-at-home orders, were also delivered by females with more compassion and empathy which is positively associated with trust (Sergent and Stajkovic, 2020). The success may also not only be about women handling the pandemic better, more that some male leaders are handling it poorly (Lewis, 2020).

The present research did not find any evidence of a difference in public trust toward male and female leaders, as seen in the distrust comparison. However, the findings indicated more offensive language in comments toward tweets authored by male leaders compared to female leaders. Aligned with this statement, in a recent study on incivility toward politicians on social media (Rheault et al., 2019), it was found that, in general, men were more targeted by uncivil messages.

### Social Media Engagement Differences

The effective use of social media for crisis communication requires engagement with followers to build relationships and trust with the public. Higher engagement with government and public authorities on social media is linked to the development of trust (Wendling et al., 2013). Transparent and timely crisis information delivered via social media is an important aspect of trust development and should guide how leaders develop strategic crisis communication plans (Wendling et al., 2013; Liao et al., 2020).

Our study found that male and political leaders followed back more accounts that followed them (i.e., friends) and liked more posts compared to females and health officer leaders. However, we did not find a significant difference between the engagement metrics of posts and replies of compared groups. Another COVID-related social media study found that engagement overall was low with government and public health related posts, even from the accounts that were active (Liao et al., 2020). The study found that the low engagement may be due to low levels of interest, trust, emotional appeal, and efficacy messages (Liao et al., 2020). The posts were mainly found to be a one-way dissemination of information about the COVID-19 situation rather than true engagement with followers (Liao et al., 2020). This is consistent with low engagement as defined in Neiger et al. (2013), where social media use is limited to information dissemination and is reported to be the stage where most public health accounts deteriorate (Neiger et al., 2013).

In the current study, we examined the effect of health authorities' gender and position on gaining trust and engagement on Twitter. However, there might be a number of confounding factors that affect the results. Extending the study in the future to consider other information such as age and location might help to explain the publics' behaviors toward health leaders more precisely. In addition, other factors such as individual personalities and the activity of authorities' accounts are likely to bias the results.

### Limitations and Future Research

There might be some limitations in the present study. We collected Twitter posts and responses corresponding to 38 administrators involved in the COVID-19 pandemic. Since the premium Twitter API was used, we were limited in adding data from a wider range of administrators' accounts. Future studies might consider including additional accounts so that the distribution of accounts will be similar among male/female, politicians/health officials and the US/Canada categories. Moreover, future studies can collect tweets in reply to politicians of each state in the US and tag them by their political views and beliefs. Comparing the sentiments regarding political attitudes can help explore trust or distrust patterns in responses to political administrators during the pandemic.

Additionally, the current study covers the first 15 months of the pandemic. Here, we divided the period of study to before and after vaccination, but our data after vaccination covered between 3 and 4 months of the pandemic. Future studies could expand the dataset to include additional data around vaccination or other critical events of the pandemic.

Finally, the current research focused on quantitative methodologies. Further research could expand the focus to include mixed methods where qualitative methods such as critical discourse analysis or content analysis can further describe the power dynamics and structural inequalities at play.

## Conclusions

In conclusion, we found that Twitter provided an environment for male and political leaders to generate more user engagement. Twitter users trusted health leaders more than political leaders. Offensive language was more often used toward political leaders' posts compared with responses to health leaders' posts. On the other hand, appreciation keywords were more used toward health officials than politicians. The present study provides insights into biases toward a specific group of leaders during emergency situations. As social media is gaining more popularity as an important source of information, it becomes critical to identify biases and combat them, in particular during important events such as the COVID-19 pandemic.

## Data Availability Statement

The raw data supporting the conclusions of this article will be made available by the authors, without undue reservation.

## Ethics Statement

Not applicable as this paper is secondary analysis of publicly available data. Research utilizing data that are both existing and public is not considered human participant research and does not require REB review (TCPS 2, Article 2.2). As long as there is no expectation of privacy, we do not require ethics approval.

## Author Contributions

SY: investigation, methodology, conceptualization, and writing—original draft. RD: conceptualization, writing—review and editing, and supervision. MM and AP: conceptualization and writing—review and editing. SS: supervision, conceptualization, writing—review and editing, and funding acquisition. All authors contributed to the article and approved the submitted version.

## Funding

This research was supported in part by the University of Guelph's Food From Thought initiative.

## Conflict of Interest

The authors declare that the research was conducted in the absence of any commercial or financial relationships that could be construed as a potential conflict of interest.

## Publisher's Note

All claims expressed in this article are solely those of the authors and do not necessarily represent those of their affiliated organizations, or those of the publisher, the editors and the reviewers. Any product that may be evaluated in this article, or claim that may be made by its manufacturer, is not guaranteed or endorsed by the publisher.
