# Supplementary material for: Trust and Engagement on Twitter During the Management of COVID-19 Pandemic: The Effect of Gender and Position
Source: Front Sociol. 2022 Apr 4;7:811589. doi: 10.3389/fsoc.2022.811589 (PMC9014168; doi:10.3389/fsoc.2022.811589)
Supplement: Supplementary file 1 [file Table_1.docx]

# Trust and Engagement on Twitter during the Management of COVID-19 Pandemic: The Effect of Gender and Position

1

## A Tables

Table A1: A list of positions and gender of collected Twitter accounts

| Position | Gender |
| --- | --- |
| Premier of Nunavut | male |
| Kansas Secretary of Health | male |
| Chief Medical Executive State of Michigan | female |
| Health Secretary of the Puerto Rico | male |
| Director at Arizona Department of Health | female |
| Premier of Alberta | male |
| State Public Health Officer and Director | male |
| Public Health Advocate | female |
| Executive Director of the Colorado | female |
| South Dakota’s Secretary of Health | female |
| Alameda County’s Health Officer | female |
| Alaska’s Chief Medical Officer | female |
| Premier of Saskatchewan | male |
| Premier of British Columbia | male |
| Premier of Quebec | male |
| Toronto’s Medical Officer of Health | female |
| Premier of Newfoundland and Labrador | male |
| Premier of Yukon | male |
| Premier of Manitoba | male |
| Nova Scotia Chief Medical Officer | male |
| Premier of Nova Scotia | male |
| Louisiana Department of Health | male |
| British Columbia Provincial Health Officer | female |
| WV State Health Officer | female |
| Canadas Minister of Health | female |
| Premier of New Brunswick | male |
| Premier of Prince Edward Island | male |
| Director of the Maine center for CDC | male |
| Alberta’s Chief Medical Officer of Health | female |
| Quebec’s Director of Public Health | male |
| NL’s Chief Medical Health Officer | female |
| NT’s Chief Public Health Officer | female |
| The Chief Public Health Officer of Canada | female |
| Commissioner for Public Health | male |
| MB’s Chief Public Health Officer | male |
| Premier of Ontario | male |
| Pennsylvania Assistant Secretary for Health2 | female |
| Premier of the Northwest Territories | female |

Table A2: A list of offensive phrases

Phrase

‘shame on you’, ‘shit’, ‘fuck’, ‘ass’, ‘bitch’, ‘crap’, ‘damn’, ‘stupid’, ‘plank’, ‘liar’, ’racist’

| close the borders. Now! |
| --- |
| Why not testing people at airport? |
| We are almost at the finish line!!! I don’t trust a word you say, why should we believe you !!!??? The weather is nice we are changing the clock there is almost no covid and we are locked up!!!! Shame on you |
| You failed to act promptly in a pandemic and you gave false information about mask to the public, which causes so many people sick and dying, if I were you I would feel ashamed and resign! |

Table A3: Distrust tweets
